# Catalogue and Price Current

**Published:** 1882-11

**Authors:** 


Catalogue and Price Current, with a Directory of the Homœopathic Physicians of New England. By Otis Clapp & Son.

This book is neatly printed and will be of use to those ordering goods by post.

